# A step forward in the diagnosis of urinary tract infections: from machine learning to clinical practice

**DOI:** 10.1016/j.csbj.2024.07.018

**Published:** 2024-07-25

**Authors:** Emilio Flores, Laura Martínez-Racaj, Álvaro Blasco, Elena Diaz, Patricia Esteban, Maite López-Garrigós, María Salinas

**Affiliations:** a Department of Laboratory, Hospital Universitario San Juan de Alicante, Carretera de Valencia, 03550 San Juan de Alicante, Alicante, Spain; b Department of Clinical Medicine, Universidad Miguel Hernandez, Elche, Spain; c Department of Emergency, Hospital Universitario San Juan de Alicante, Carretera de Valencia, 03550 San Juan de Alicante, Alicante, Spain

**Keywords:** Urinary Tract Infection, Emergency department, Urinalysis, Laboratory Medicine, Clinical Decision Support Systems, Machine learning

## Abstract

**Objectives:**

Urinary tract infections (UTIs) are common infections within the Emergency Department (ED), causing increased laboratory workloads and unnecessary antibiotics prescriptions. The aim of this study was to improve UTI diagnostics in clinical practice by application of machine learning (ML) models for real-time UTI prediction.

**Methods:**

In a retrospective study, patient information and outcomes from Emergency Department patients, with positive and negative culture results, were used to design models – ‘Random Forest’ and ‘Neural Network’ – for the prediction of UTIs. The performance of these predictive models was validated in a cross-sectional study. In a quasi-experimental study, the impact of UTI risk assessment was investigated by evaluating changes in the behaviour of clinicians, measuring changes in antibiotic prescriptions and urine culture requests.

**Results:**

First, we trained and tested two different predictive models with 8692 cases. Second, we investigated the performance of the predictive models in clinical practice with 962 cases (Area under the curve was between 0.81 to 0.88). The best performance was the combination of both models. Finally, the assessment of the risk for UTIs was implemented into clinical practice and allowed for the reduction of unnecessary urine cultures and antibiotic prescriptions for patients with a low risk of UTI, as well as targeted diagnostics and treatment for patients with a high risk of UTI.

**Conclusion:**

The combination of modern urinalysis diagnostic technologies with digital health solutions can help to further improve UTI diagnostics with positive impact on laboratory workloads and antimicrobial stewardship.

## Graphical Abstract

**Figure:**
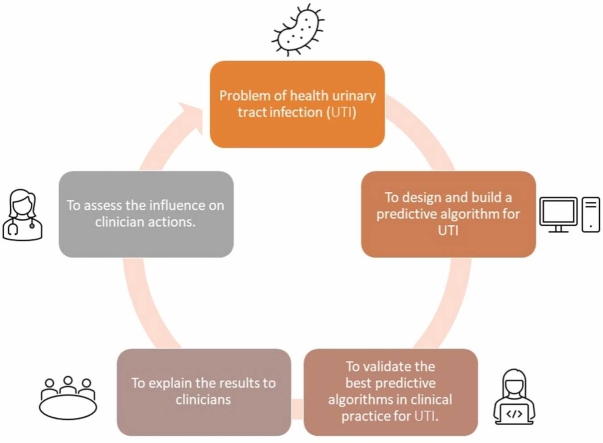

## 1 Introduction

Urinary tract infections (UTIs) represent one of the most frequently diagnosed infectious disorders and are more prevalent in women, with more than 50 % of females facing at least one UTI during their life [1]. Depending on the site of infection, UTI can be classified as upper UTI (pyelonephritis/nephritis) or lower UTI (urethritis, cystitis, prostatitis). Depending on the presence of comorbidities, pregnancy or the sex and age of the patient, UTI can be further differentiated into uncomplicated and complicated UTI. The heterogeneity of the associated symptoms can include oliguria, pollakiuria, dysuria, abdominal pain, fever and others. This reflects the diverse expression of the severity of UTI, which ranges from asymptomatic bacteriuria to septic shock and often hinders correct UTI management [2].

UTIs are a major burden not only for individual patients but also for healthcare systems. With more than 400 million confirmed cases annually [3], UTIs cause a significant workload and substantial expenditures for healthcare systems and economies. In terms of costs, UTIs pose a significant social and personal burden. Recurrent infections are associated with increased absenteeism from work and physician visits. Furthermore, UTIs constitute a huge burden on healthcare systems as they are a frequent reason for hospitalization [1]. Among the different healthcare settings within hospitals, emergency departments (EDs) handle most UTI patients. In the United States, for example, UTIs result in more than 3 million ED visits per year, and also in estimated costs of up to $2 billion US annually [4]. The increase in the geriatric population and its age-related diseases, as well as the costs related to the inappropriate prescription of antibiotics, contribute to this high economic burden.

Increasing resistance to antibiotics among uropathogenic bacteria has been declared a serious threat to public health by the World Health Organization (WHO) [5] and UTIs have already become the fourth most common cause of death in the context of antimicrobial resistance [6]. Since UTIs account for around 15 % of all antibiotic prescriptions in Europe and the United States [7], urinary pathogens are a relevant cause of the current antimicrobial resistance emergency, demanding the need for appropriate antimicrobial stewardship [8]. The overuse of antibiotics could decrease if the diagnostic error of UTIs is acted upon; only in EDs, this error is estimated to range from 30 to 50 % [9], [10], [11]. As previously mentioned, symptoms and physical findings are heterogeneous and laboratory results are often not as accurate as expected. Indeed, *Meister et al.* found that the only predictor of UTIs with enough specificity was a positive nitrite result [12]. Undoubtedly, UTI diagnosis is hampered by urine cultures, which have been (and still are) part of the diagnostic gold standard but are time-consuming as result availability for urine cultures requires at least 24–48 h after inoculation. This is quite a long-time span, considering that the majority of suspected UTIs turn out to be negative. For these reasons, culture results are rarely available in real time to influence therapeutic decisions for a new UTI and cause empiric antibiotic prescriptions, often in absence of diagnostics at all [13], [14].

In this context, there is a need for the design and development of clinical decision support systems (CDSS) that are integrated with artificial intelligence (AI) tools and support physicians in accurate decision making. These systems are defined as “any process to improve health-related decisions and actions with pertinent, organized clinical knowledge and patient information to improve healthcare and healthcare delivery” [15], [16] and their implementation into different healthcare settings, e.g. the clinical laboratory, can be considered a revolution [17].

Health information technologies (HIT) have advanced over the last decades and have changed the role of laboratories within laboratory medicine from traditional laboratories to “leading” laboratories that are focused on the patient and do not just intervene in clinical decision-making but also make clinical decisions. Currently, laboratory medicine contributes to most medical decision-making processes; however, it is still a challenge to include these novelties in clinical workflows [17].

We hypothesized that in our hospital ED, the application of ML models collecting demographic data, urine test strips and flow cytometry could potentially improve the UTI diagnostics and influence daily clinical practice, aiming to provide ED physicians with an algorithm for real-time UTI prediction. Therefore, we first designed and built predictive models to reflect the risk of contracting a UTI in ED patients using AI. Afterwards, we validated the best predictive models in real clinical practice, and finally, we assessed the impact of these models on physician behaviour by measuring changes in antibiotic prescriptions and urine culture requests.

## 2 Material and methods

### 2.1 Design

**Fig. 1:**
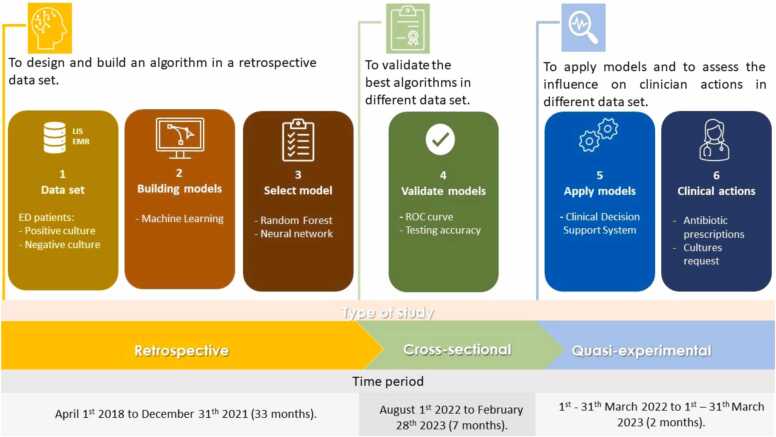
– Timeline diagram of the three types of studies conducted and their respective aims.shows the consecutive steps of the study and a timeline diagram. summarizing study periods and the aims of the three mentioned investigations.

We designed a mixed and sequential study from April 2018 to March 2023. This single-centre study was composed of a retrospective observational approach, which was used to design and build UTI predictive models. Then, a cross-sectional study was set up to validate the best predictive models in clinical practice. Finally, a quasi-experimental study assessed the influence of these models on clinician’s actions and behaviour. Fig. 1 shows a timeline diagram, summarizing study periods and the aims of the three mentioned investigations.

### 2.2 Laboratory and hospital characteristics

Our clinical laboratory is in a 396-bed suburban university community hospital (Hospital Universitario San Juan de Alicante - Sant Joan d’Alacant) that serves inhabitants from the Department of Health (DH) region “Alicante - Sant Joan d’Alacant”. The 238,521 inhabitants (report from year 2022) of this DH region are served by the central hospital and nine Primary Care (PC) centres. The DH region is integrated into the public health network of the Valencian Community, Spain.

Our laboratory receives samples from inpatients, ED patients, and PC patients collected at PC centres within the region. Laboratory requests are demanded through an electronic system, the Computerized Physician Order Entry (CPOE), which offers ED physicians certain tests on a stat basis.

Our laboratory has implemented a laboratory information system (LIS) (Gestlab, v2022.4.8, Alicante, Spain) and a clinical decision support system (CDSS) as a rule engine (AlinIQ CDS v8.2, Abbott, Chicago US) to manage pathways and actions. CDSS was developed through iterative consultation with multiple clinical stakeholders [18]. The urinalysis laboratory workflow is completely automated: all urine samples are processed by test strip readers and when results of haemoglobin peroxidase, leukocyte esterase or nitrite are positive, samples are subjected to urinary flow cytometry that is done by a totally integrated analyser.

### 2.3 Participants and variables

ED patients with a suspected UTI and a request for a urine culture were defined as “cases”. Pregnant women were not selected and patients aged < =12 years were excluded. Cases with contaminated cultures were also rejected according to the criteria of the European Urinalysis Guidelines of 2000 [19].

Predictor variables included demographic information (age and sex), body temperature and laboratory test results (urine dipstick test and urinalysis by flow cytometry). All data has been stored in encrypted files and did not contain sensitive or personally identifiable information.

The primary outcome for all analyses was the presence of a positive urine culture defined by $10^{4}$ colony forming units (CFU)/ML, a threshold pre-established by the laboratory of our healthcare system to report positive results based on the standards of previous studies [4]. The study was conducted in accordance with the Declaration of Helsinki and approved by the Institutional Review Board of the University Hospital of San Juan de Alicante - Sant Joan d’Alacant.

### 2.4 Retrospective study

To build a predictive algorithm for UTIs, we obtained a LIS dataset over a 33-month period (1st April 2018 to 31th December 2021). Data was randomly allocated (70 %/30 %) into independent partition sets for training (creation of models) and validation (model selection). The two sets of random samples were stratified by the target variable. Data were pre-processed according to previously described methods [4], [20]. Categorical variables were included in the models in their current categorical form. All continuous variables were standardized by removing the mean and scaling to unit variance using Python toolbox (v3.9.0; Python Software Foundation, USA; sklearn.preprocessing.standardScaler). The primary goal of our modelling approach was to find patterns/relations in data between the dependent variables (UTI) and the independent variables (predictor variables). To predict the risk of UTI occurrence in the ED, we developed two models using different ML models: Neuronal Network (NN) and Random Forest (RF). Models were trained on the training partition and the validation partition was used to assess all models previously created using the training data. Assessment and comparison of models was based on the receiver operating characteristic curve index (ROC index). SAS Enterprise Miner 14.3 was used for mode development.

### 2.5 Cross-sectional study

We compared the two predictive models (NN and RF) in clinical practice during a study period of seven months (1st August 2022 to 28th February 2023). After selecting cases with the same criteria as those applied to the retrospective study, we compared the results of each predictive algorithm separately and the action of both models together (RF+NN). RF+NN were analyzed when their results were coincident. In cases of discrepancy, the data were considered missing. Assessment of models divided by gender were based on the ROC index, diagnostic testing accuracy and parameters constituting confusion matrices as shown in Fig. 1.

The communication developed in Python was deployed in the CDSS integration engine. This algorithm requires a set of laboratory results per patient. Whenever this dataset is available, it is sent to the Python script to receive the results via the CDSS. Once the data is collected, the integration engine creates the dataset and transforms it into the format required by the CDSS system. Subsequently, the results are transferred to the LIS. To improve data processing in real time, we divided the models by gender.

### 2.6 Quasi-experimental study

The intervention was designed during two meetings between laboratory professionals and ED physicians. The cross-sectional study results were showed and analysed, and the implementation of a new workflow was decided, as follows.

When a urinalysis was requested from the ED, if the dipstick test was positive (haemoglobin peroxidase, leukocyte esterase or nitrite), we proceeded with flow cytometry. Subsequently, an automated process calculated the algorithm for both ML models. An automatic comment of "low risk of UTI" or "high risk of UTI" would appear in the laboratory report only when both models coincided. We counted the number of cases with the same prediction in the two ML models, which were consequently reported through a comment in the laboratory report, and those in which there was no coincidence between both models, and the prediction was not reported.

It was also decided to assess the impact of the intervention in a new data set by comparing it with a pre-intervention period when the UTI prediction was calculated but not reported, and consequently unknown by the ED clinician as shown in Fig. 1. Through a review of patients' electronic medical record (EMR), the actions taken by the ED clinician regarding antibiotic treatment, when low and high-risk predictions, were acknowledged. To determine the total number of patients that were correctly treated in both periods, the ratio of patients for whom ED clinician make the correct actions (treated when at high risk plus not treated when low risk) to total number of patients was calculated. Additionally, the action taken by the ED clinician regarding urine culture requests, when low and high-risk predictions were assessed. The first consecutive cohort (pre-intervention period, March 2022) was compared with a second consecutive cohort (post-intervention period, March 2023).

The sample size was calculated from data on UTI prevalence in Spanish Hospital EDs published in a prior study and assumed a frequency of 3.2 % [21]. A 99 % confidence level indicated a minimum cohort size of 268 cases for both periods, considering our population.

### 2.7 Laboratory methods

The fully automated urine test strip analyser UC-3500 (Sysmex Corporation, Kobe, Japan) was used for semi-quantitative measurement of specified analytes in human urine and commercially available control materials according to the manufacturer's instructions. Test strips (Meditape UC-9A, Sysmex Corporation, Kobe, Japan) were used in this study. These strips include reagent pads for ordinal scale reporting of urobilinogen, glucose, protein, haemoglobin peroxidase, nitrite, bilirubin, ketone, leukocyte esterase and pH. The instrument is equipped with a reflective photometry unit and reagent strips are scanned with a colour complementary metal oxide semiconductor detector (CMOS) that takes reflectance readings from the reagent strip. The light reflected off the reagent pad is used to measure the concentration of a substance present in the urine. A high concentration of an analyte corresponds to a low reflectance. The reflectance value, expressed as a percentage within a range from 100 % (white) to 0 % (black), is inversely related to the concentration of the analyte. To cover the whole measuring range with good linearity, two overlapping reflectance ranges are installed for the parameters pH, protein, glucose, bilirubin and ketones [22].

Pre-screened urine samples subjected to urinalysis by flow cytometry were analysed for all parameters available in the Sysmex UF-5000 instrument (Sysmex Corporation, Kobe, Japan), which is the latest generation urine and body fluid analyser from Sysmex. In the instrument, cells are counted and classified by analysing forward scattered light, side scattered light, side fluorescent light and depolarized side scattered light. The analyser can discriminate and count 17 diagnostic parameters of cells and formed elements in urine. We selected seven parameters: red blood cells (RBO), white blood cells (WBC), bacteria (BACT), crystals (XTAL), squamous epithelial cells (EC), hyaline cast (CASTS) and yeasts (YLC). The system employs fluorescence flow cytometry technology, where urinary particles are stained by channel-specific fluorochromes for nucleic acids (CRch) and for surface structures (SFch). The stained particles are separated by hydrodynamic focusing before passing through the laser beam, a new blue semi-conductor laser with 488 nm wavelength.

For all the samples, a standard quantitative urine culture was performed by inoculating 1 μL of well-mixed urine specimen using a calibrated loop, both on a non-selective Columbia Blood agar plate (BioMerieux, France) supporting the growth of UTI pathogens, and on a selective McConkey agar (BioMerieux, France). The plates were incubated aerobically at 35–37 °C for 18–24 h. Results were expressed as the number of colonies forming unit per millilitre (CFU/ML).

### 2.8 Data analysis

Descriptive statistics were presented for variables are non-parametrically distributed as median and IQR (percentile 25–75) and percentages for continuous data and categorical data. The comparative study by two percentages was done by the χ2-test. The differences between groups were assessed using the Wilcoxon signed-rank test. A two-sided p ≤ 0.05 rule was used as the criterion for rejecting the null hypothesis of no difference. The results of the predictive models were compared with the results of urine cultures using ROC curve analysis and diagnostic testing accuracy using the following parameters: sensitivity, specificity, accuracy and positive and negative likelihood ratios (LR +/). The results of RF were dichotomic (negative or positive) and NN value was quantitative (greater than 0.49 was considered positive). Parameters constituting confusion matrices: true positives (TP), false positives (FP), true negatives (TN), false negatives FN, positive predictive value (PPV) and negative predictive value (NPV) were calculated with respect to the reference urine culture test. ROC curve was accompanied by their respective 95 % confidence interval and p values.

## 3 Results

### 3.1 Retrospective analysis

**Fig. 2–:**
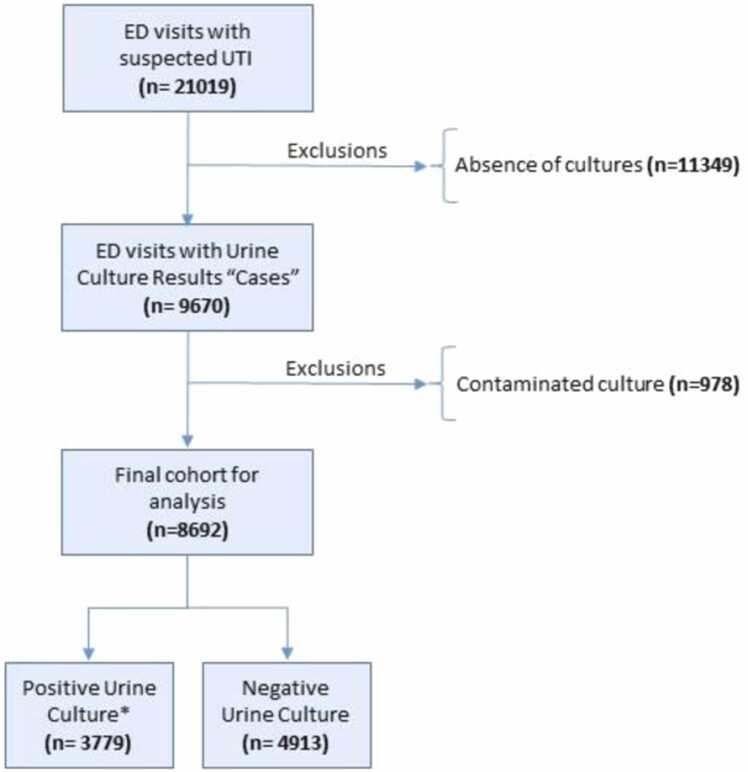
Work-flow diagram: retrospective analysis from Emergency Department (ED).

We trained and tested two different predictive models. Over a 33-month study period, 8692 cases were registered and included into a final data set. The workflow diagram for this study approach is displayed in Fig. 2. The patient population constituting the training data set was characterized by a median age of 72;33 years with a female proportion of 57.4 %.

**Fig. 3:**
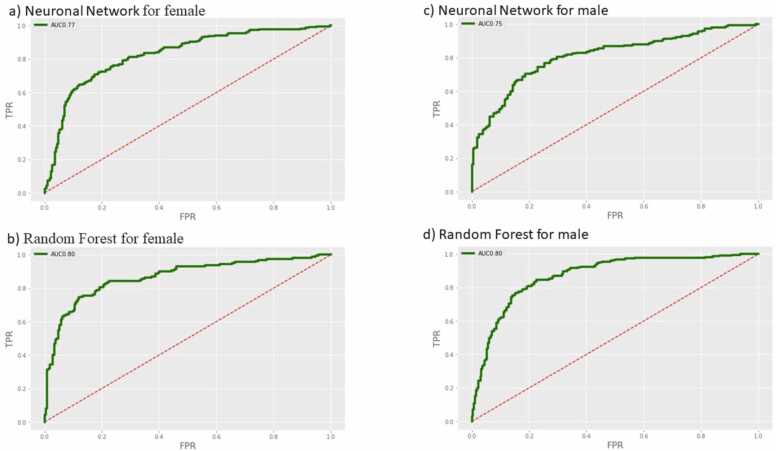
– Work-flow diagram: retrospective analysis from Emergency Department (ED). Study period: 33 months [01/04/2018 – 31/12/2021]. retrospective analysis from HUSJA Emergency Department (ED). Study Time Period: 33 months [01/04/2018 – 31/12/2021]. We excluded patients due to the lack of gold standard and due to contaminated culture. *Positive urine culture defined by: >$10^{4}$ colony forming units/ML. UTI: Urinary tract infection ROC indices for the NN and RF models by gender.TPR: True positive rate; FPR: False positive rate.

Test characteristics for the final two ML models, RF and NN, were calculated in consideration of the sex of the patients as shown in Fig. 3. The ROC indices ranged from 0.77 [0.74–0.80] (p values <0.05) NN to 0.80 [0.77–0.83] (p values <0.05) RF and 0.75 [0.72–0.79] (p values <0.05) NN to 0.80 [0.77–0.84] (p values <0.05) RF for female and male patients, respectively. When assessing the entry model, no significant difference can be observed for the ROC indices for the two chosen models.

### 3.2 Cross-sectional analysis

**Fig. 4:**
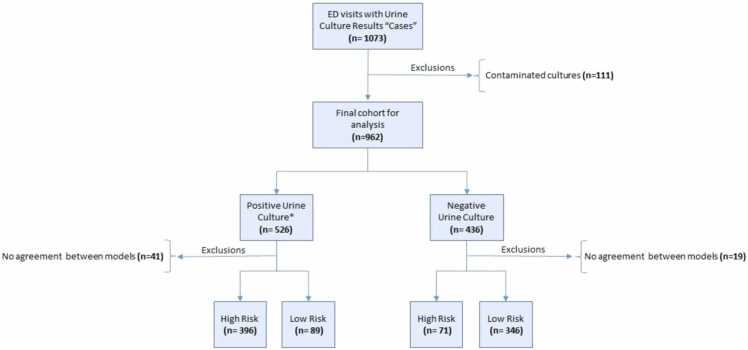
Work-flow diagram: cross-sectional analysis from Emergency Department (ED).Work-flow diagram: cross-sectional analysis from Emergency Department (ED). Study period: 7 months [01/08/2022–28/02/2023]. *Positive urine culture defined by: > 10^4 colony forming units/ML.

We investigated the performance of the predictive models in clinical practice. Within a 7-month study period, 962 cases were registered and included into a final dataset. The corresponding workflow diagram for this study approach is displayed in Fig. 4. The median age of the cases constituting the validation dataset was 73;29 years with a female proportion of 55.8 %. No significant differences were reported between the datasets. ROC index, diagnostic testing accuracy and parameters constituting confusion matrices were calculated as shown in Table 1.

**Table 1 –:** Diagnostic Testing Accuracy.

**Models**	**TP**	**FP**	**TN**	**FN**	**AUC** **(95 % CI)**	**Sensitivity (95 % CI)**	**Specificity (95 % CI)**	**Accuracy** **(95 % CI)**	**LR + (95 % CI)**	**LR - (95 % CI)**	**PPV** **(95 % CI)**	**NPV** **(95 % CI)**
**Random Forest model**												
Women	*247*	*41*	*179*	*70*	0.81[0.78 −0.85]*	*78[73–82]%*	*81[76–86]%*	*79[77–83]%*	4.11[3.15 −5.54]	0.27[0.22 −0.34]	*86[82–89]%*	*86[82–89]%*
Men	*177*	*39*	*177*	*32*	0.85[0.81 −0.89]*	*85[79–89]%*	*82[76–87]%*	*83[79–87]%*	4.72[3.51 −6.27]	0.18[0.14 −0.26]	*82[77–86]%*	*85[79–87]%*
**Neural Network model**												
Women	*235*	*39*	*181*	*82*	0.84[0.81 −0.88]*	*74[69–79]%*	*82[77–87]%*	*78[74–81]%*	4.18[3.12 −5.60]	0.31[0.26 −0.38]	*86[82–89]%*	*69[65–73]%*
Men	*174*	*42*	*174*	*35*	0.88[0.84 −0.91]*	*83[78–88]%*	*81[75–86]%*	*82[78–86]%*	4.37[3.24 −5.65]	0.21[0.15 −0.28]	*81[76–85]%*	*83[79–87]%*
**Combine Neural Network and Random Forest models**												
Women	*228*	*36*	*176*	*63*	0.81[0.78 −0.85]*	*78[73–83]%*	*83[77–88]%*	*80[77–84]%*	4.60[3.41 −6.25]	0.26[0.21 −0.33]	*86[82–90]%*	*74[69–78]%*
Men	*168*	*35*	*170*	*26*	0.85[0.81 −0.89]*	*87[81–91]%*	*83[77–88]%*	*85[81–88]%*	5.07[3.73 −6.89]	0.16[0.11 −0.23]	*83[78–87]%*	*87[82–90]%*

Performance values obtained for each predictive models and RF+NN classifiers in the final dataset of the cross-sectional cohort. TP=true positives; FP=false positives; TN=true negatives; FN=false negatives; AUC=area under the curve; CI=confidence interval; LR+ /-=positive and negative likelihood ratios; PPV= positive predictive value; NPV negative predictive value.* p values < 0.01. The model result of RF was dichotomic (negative or positive) and NN value were quantitative (greater than 0.49 was considered positive).

The best sensitivity value was observed for men with coinciding RF and NN, showing a maximum of 0.87. Regarding the specificity, the highest value was also associated with cases with coinciding RF and NN, but there was no difference between women and men. The same applies to accuracy; RF and NN men achieved the best value: 85 %. LR + /- also followed the trend: LR + /- with best value were attributed to men where the decision of both models, RF and NN, was the same: a LR+ of 5.07 and a LR- of 0.16 was obtained.

### 3.3 Quasi-experimental analysis

Coincidence between both ML models were measured in 820 consecutive ED cases after the intervention was implemented. In 20 cases there was no coincidence, so it was informed in 97.6 % of the cases. To assess the impact of the intervention, we compared 600 consecutive ED cases in pre-intervention and post-intervention period. With regards to sex, pre-intervention period 370 (61.7 %) of women vs. post-intervention period 353 (58.8 %) of women. With regards to age, pre-intervention period 58;39 years old vs. post-intervention period 63;39 years old, there were no significant differences between datasets.

**Fig. 5:**
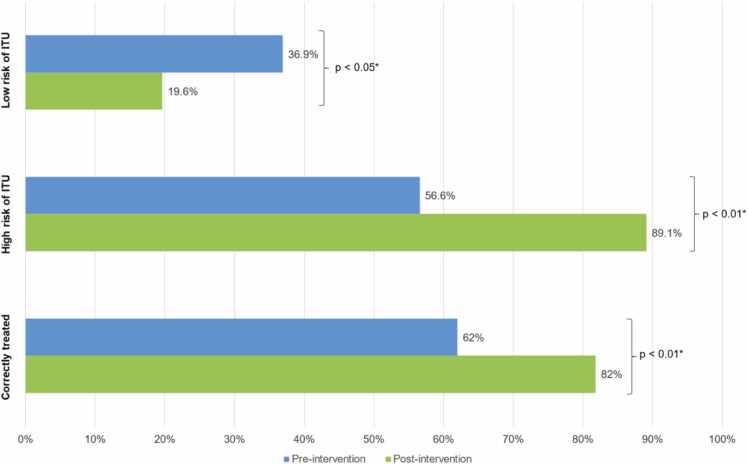
Changes in antibiotics prescription: pre-intervention vs. post-intervention.Physicians’ behaviour regarding antibiotic prescriptions and cases that were correctly treated from the Emergency Department before (pre-intervention) and after (post-intervention) implementation of the new diagnostic process. * A two-sided p ≤ 0.05 rule was used as the criterion for rejecting the null hypothesis of no difference. UTI: Urinary tract infections.

For the prescription of antibiotics, a change in the clinical practice has been observed as shown in Fig. 5. When the models reported a low risk for UTI (501 pre-intervention vs. 499 post-intervention) the number of cases who received antibiotics were higher in pre-intervention (n = 185) period than in post-intervention (n = 98) period. ED physicians prescribed fewer antibiotics in ‘low risk of UTI’. When the models reported a high risk for UTI (99 pre-intervention vs. 101 post-intervention) the number of cases who received antibiotics were higher in post-intervention (n = 90) period than in pre-intervention (n = 56) period. ED physicians prescribed more antibiotics in ‘high risk of UTI’. The total number of cases that were correctly treated were 372 in pre-intervention and 491 in post-intervention period.

**Fig. 6:**
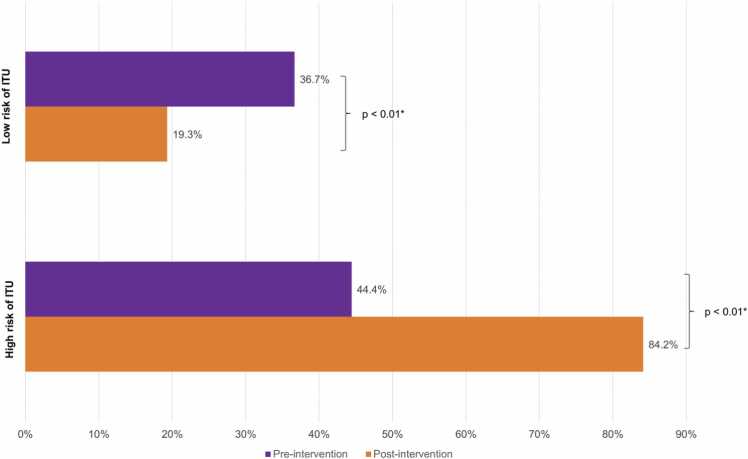
Changes in urine culture request: pre-intervention vs. post-intervention.Physicians’ behaviour regarding urine culture requests from the Emergency Department before (pre-intervention) and after (post-intervention) implementation of the new diagnostic process. * A two-sided p ≤ 0.05 rule was used as the criterion for rejecting the null hypothesis of no difference. UTI: Urinary tract infections.

Changes in the total number of urine culture requests were compared between pre- and post-interventions (Fig. 6). Highlighting a decrease in urine culture requests when the models stated a low risk of UTI (220 pre-intervention vs. 126 post-intervention). An upward trend can also be observed when models reported a high risk of UTI: ED physicians ordered more urinary cultures (44 pre-intervention vs. 85 post-intervention).

## 4 Discussion

This is the first study to report an intervention for the real-time prediction of the likelihood of UTIs in an ED setting. The objective was to predict positive urine culture results and to rule-out UTIs using urinalysis tests with real-time access to patient data. This intervention allowed ED physicians to prescribe antibiotics with greater accuracy, as verified through the subsequent review of EMR. Furthermore, the result was achieved by means of ML models: our system learned which pattern of urinalysis tests results and which body temperature predicted or ruled out most UTI diagnoses.

In recent years, AI has already been used to train and validate predictive models for the diagnosis of UTIs. Authors primarily put their efforts into retrospective studies, which are based on the compilation of large databases to develop models through ML [23]. *Moller et al.* collected approximately 300,000 past adult hospital admissions with their respective demographic information, laboratory results, antibiotic treatments, prior medical history records and some other clinical data to train and evaluate five different ML models [24]. *Mingkuan et al.* described another retrospective approach; they screened 26 different laboratory data variables from more than 500,000 cases to train and validate six ML models [25]. Another recent example is attributed to *Burton et al.*, who analysed a total of 212,554 past urine reports to verify if ML models (independently or in combination) outperformed a concrete heuristic model [26]. Even though good diagnostic testing accuracy results are found through the literature, the deployment of AI tools is not always easy and lacks reproducibility [27]: big amounts of data are needed and there is no consensus on statistically significant variables.

Urine samples from ED patients, which are random urines, are the only ones that arrive at our laboratory in fresh conditions. This is because most of the samples we receive for urinalysis in our laboratory are collected at home, taken to the PC centre, and then delivered to the hospital for analysis or directly to the laboratory in the hospital, as is the case in most laboratories in Spain [28]. This implies that urine particles remain intact during the process. The pre-analytical conditions are perfect for the urine particle to remain intact until analysis.

On the other hand, technology for urinalysis, a set of urine tests, has greatly improved and grown in the last decades. The improvement spans from the simple reporting of basic semi-quantitative tests, which can now even be used as a screen to avoid tests quantification under certain conditions [29], to new research parameters. In fact, urinalysis is processed through highly modern automated analysers for particle recognition and tracks that give urinalysis results increasing reliability. These new advancements in urinalysis, coupled with the availability of IT digital solutions, allow laboratory medicine to make an impact beyond the laboratory walls and, in agreement with ED physicians, improve the diagnosis and treatment of UTI patients.

The implementation of this intervention has three important consequences or benefits. Patients will leave the ED with a better chance of receiving the correct UTI diagnosis and treatment. As discussed, UTIs result in significant distress to patients, impact their quality of life [30] and can lead to severe complications. In addition, within the community, patients older than 65 years with a UTI diagnosis are reported to be at significantly increased risk of bloodstream infection and death within 60 days when antibiotic treatment was not prescribed or was delayed [31]. Second, with the implementation of this invention, we reduce inappropriate antibiotic prescriptions, and thereby foster antimicrobial stewardship, with the aim of reducing resistance to antibiotics, a severe global problem and one of the biggest threats to global health, food security and development today. It seems that this condition could be higher in our health department due to the presence of a very old population, that could be attributed to our location, situated by the Mediterranean Sea, and the favourable climate that attracts people from all over the world for retirement. In fact, with the implementation of this invention, we reduce inappropriate antibiotic prescriptions, and thereby foster antimicrobial stewardship, with the aim of reducing resistance to antibiotics, a severe global problem and one of the biggest threats to global health, food security and development today [32]. Third, we perform this intervention without any additional costs by simply combining the results of the traditional and commonly used urinalysis test, real-time access to body temperature through digital health solutions that are now within our reach in laboratory medicine.

Our study has several limitations. Firstly, we had to exclude cases with suspected UTI from the model creation due to the absence of the gold standard (culture was not requested). This exclusion may have introduced a source of bias in the model construction. Secondly, the type of urine sample used, which is a random urine rather than a clean-catch midstream urine. The EFLM European Urinalysis Guideline, Update 2023 [33], defines random urine as a "portion of single voided urine without defining the volume, time of the day, or detail of patient preparation, usually the unavoidable case in acute situations, associated with many false negative and some false positive results." These acute situations include ED patients. Since random urine is the only available sample we have at our disposal, and because our ML models have been trained using this type of sample, they may be overwhelmed by it. A new limitation could be a study bias, because we only selected cases with a positive dipstick test and requested culture. Therefore, it makes the pre-test probability very high "per se", and the model may have an overestimated performance in these cases. Another limitation is that we could have not clearly explained that our risk assessment model, which predicts the likelihood of developing an infection, is different from a diagnostic model, which identifies the presence of an infection. Finally, while our model performs well in our specific setting, having been validated through the review of each patient's medical record, it may require adjustments to be extrapolated effectively to another environment.

Deploying this model for diagnosing urinary tract infections in real clinical settings offers the potential to significantly enhance community care. By upscaling and generalizing its use, healthcare providers can leverage machine learning to quickly and accurately identify infections, leading to more timely treatments and improved patient outcomes. This approach could be seamlessly integrated into existing clinical workflows, making advanced diagnostic capabilities accessible to a broader population.

The main strength of this study is to show the process from ML to clinical practice as shown in Fig. 1. Any laboratory equipped with modern urinalysis technology and digital solutions can develop the methodology outlined in our study and implement real-time UTI prediction for ED patients. It is important to highlight that urine from ED patients is highly suitable for urinalysis testing, as it is fresh random urine. Moreover, advancements in both laboratory analysers and digital solutions have significantly improved in recent years. Our model, while simple, can yield impactful results in improving patient outcomes. It serves as another example of the potential of laboratory transformation, transitioning from a traditional role of merely providing data for clinical decisions to actively participating in clinical decision-making processes. This transformation aims to enhance patient outcomes and redefine the laboratory's image and role, transforming it from a data provider to a hub for clinical decision maker hub [17] that does not only test but actively improves and saves patients’ lives [34].

## 5 Conclusion

A strategy grounded in ML and digital health solutions plays a pivotal role in enhancing the clinical work-up of UTIs in the ED and promoting the appropriate use of antibiotics. As mentioned earlier, the modern clinical laboratory transcends the confines of traditional laboratory walls and actively engages in the broader healthcare landscape. It operates within a diverse and dynamic stakeholder environment, contributing to healthcare delivery by fulfilling its mission of disease prevention, diagnosis, monitoring, and treatment. Moreover, it aspires to maximize benefits for patients, clinicians, and payers alike. [17].

## CRediT authorship contribution statement

**Patricia Esteban:** Investigation, Formal analysis. **Maite Lopez-Garrigos:** Visualization, Validation. **María Salinas:** Writing – review & editing, Writing – original draft. **Emilio Flores:** Writing – review & editing, Writing – original draft, Visualization, Validation, Supervision, Data curation, Conceptualization. **Martínez-Racaj Laura:** Investigation, Funding acquisition, Formal analysis, Data curation. **Alvaro Blasco:** Software, Resources, Project administration, Methodology, Conceptualization. **Elena Diaz:** Writing – review & editing, Writing – original draft, Supervision.
